# The efficacy of custodiol as blood cardioplegia for myocardial protection in adult cardiac surgery

**DOI:** 10.1186/1749-8090-10-S1-A87

**Published:** 2015-12-16

**Authors:** Ihab Mohamed Yehya Moursi, Shaban Halawa

**Affiliations:** 1 Cardiac Surgery Department, Prince Sultan Cardiac Center, King Khaled Hospital, Al Kharj 11942, Saudi Arabia; 2 Cardiac Anesthesia Department, Prince Sultan Cardiac Center, Al Hofuf 31982, Saudi Arabia

## Background/Introduction

Custodiol cardioplegia is attractive method of myocardial protection, as a single dose provides a long period of preservation. Despite widespread use in Europe, the available data confirming its efficacy is little compared with conventional methods of cardioplegia. Custodiol solution is administered in a single-dose, allowing the operation to be done continuously. This is an advantage over other cardioplegic solutions that may have to be re administered every 20-30 minutes.

## Aims/Objectives

Is to compare the efficacy of Custodiol to standard Plegisol blood cardioplegia in adult cardiac surgical cases

## Method

This study was a single-center retrospective review of prospectively collected data. Adult cardiac surgery cases performed between January 2011 and August 2013 using Custodiol^® ^were compared to cases using standard Plegisol blood cardioplegia. The endpoints of intra-operative and post-operative were compared including 30-day mortality, hospital readmission, prolonged mechanical ventilation time, and renal failure.

## Results

Of the 100 cases identified, 40 cases used Custodiol and 60 used blood Cardioplegia. Demographics data were similar in both groups with a mean patient age of 60 ± 14.1 years for Custodiol and 66 ± 10 years for blood cardioplegia. The average cardiopulmonary bypass time for Custodiol and blood cardioplegia was 122 ± 60 and 135 ± 54 minutes respectively. The Custodiol group had a greater incidence of prolonged ventilation (>24 hours), 20% versus 15 % respectively, and this approached statistical significance with a p value of (0.05). Intra-operative blood usage was significantly higher in the Custodiol group compared to the blood cardioplegia group, with 44% of patients receiving fresh frozen plasma during the operation compared to only 25% in the blood cardioplegia group (p = 0.005). There is no statistically significant difference in 30-day mortality, hospital readmission, and renal failure.

## Discussion/Conclusion

Custodiol is effective for myocardial protection with distinct advantage of long-term ischemic tolerance although the use of custodiol increase the need of fresh frozen plasma during the perioperative period when compared to blood cardioplegia.

